# Unveiling the metabolic fate of monosaccharides in cell membranes with glycomic and glycoproteomic analyses†
†Electronic supplementary information (ESI) available: Supplementary information includes six figures. See DOI: 10.1039/c9sc01653h


**DOI:** 10.1039/c9sc01653h

**Published:** 2019-06-11

**Authors:** Gege Xu, Maurice Wong, Qiongyu Li, Dayoung Park, Zhi Cheng, Carlito B. Lebrilla

**Affiliations:** a Department of Chemistry , University of California , One Shields Avenue Davis , Davis , CA 95616 , USA . Email: cblebrilla@ucdavis.edu; b Department of Biochemistry and Molecular Medicine , University of California , Davis , CA 95616 , USA; c Foods for Health Institute , University of California , Davis , CA 95616 , USA

## Abstract

Cell membrane protein glycosylation is dependent on the metabolic state of the cell as well as exogenous nutrients available. Although the metabolism and interconversion of monosaccharides have been well-studied, their incorporation into cell surface glycans and their corresponding glycoproteins remains relatively unknown. In this study, we developed a method to investigate quantitatively the incorporation pathways of dietary saccharides into specific glycans and glycoproteins on the cell membrane by treating intestinal Caco-2 and hepatic KKU-M213 cells with ^13^C-labeled monosaccharides and characterizing the resulting cell surface glycans and glycopeptides by LC-MS/MS. Time-course studies using uniformly labeled glucose revealed that the rate of incorporation was both glycan-specific and protein-dependent. Comparative studies using different dietary saccharides and multiple cell lines revealed the variance of monosaccharide utilization and interconversion in different tissues and organisms. The robust isotope-labeling and glycan profiling methods can provide a useful tool for differentiating glycosylation pathways and enhance the understanding of how dietary sugar intake affects health.

## Abstract 2

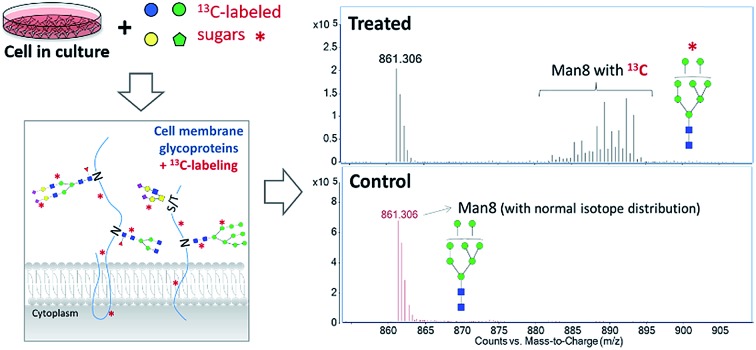
Utilizing glycomic and glycoproteomic approaches to elucidate and quantify the different patterns of sugar usage in different cell lines.

## Introduction

The utilization of monosaccharides through cell metabolism is a fundamental process in diet and nutrition.^1^–^4^ Previous studies have focused primarily on glycolysis and the fate of glucose through now established basic cellular pathways. Both stable and radioactive isotope labeling have been used to determine with great detail the fate of specific components of glucose and its metabolic products.^5^,^6^ Less is known about how monosaccharides are utilized to produce glycoconjugates. Human proteins are largely glycosylated, with glycans refining and defining the protein functions. Monosaccharides like amino acids, can be directly incorporated into proteins. While the incorporation of amino acids is well known,^7^ incorporation of monosaccharides into glycans is relatively unexplored. Only recently, comprehensive glycomic and glycoproteomic profiling methods have been made available to allow isotopic enrichment studies of glycans.

As one of the key components of plasma membrane, cell surface glycoproteins are known to be closely related to basic functions of the cell such as proliferation, differentiation, and cell–cell and cell–microbe interactions.^8^–^11^ Biosynthesis of cell membrane glycoproteins requires both glycan-processing enzymes including glycosyltransferases and glycosidases, and activated nucleotide sugars as glycosylation precursors.^12^,^13^ Although these monosaccharide donors can be salvaged from degraded glycans within the cell, the major source of glycosylation precursors is the external sugars that are imported into the cell.^14^,^15^ Therefore, glycosylation pattern on the cell surface is greatly dependent on both the metabolic state of the cell and the types of exogenous nutrients available.^16^–^18^ Monosaccharides such as glucose and fructose are the major carbon sources for most organisms and can participate in the biosynthesis of both the peptide and the glycan parts of glycoproteins.^19^–^21^ However, our recent study has shown that the use of certain sugars such as fructose and galactose as a carbon source can introduce significant changes on membrane protein glycosylation and thereby affect epithelial cell functions.^16^ Other studies have also indicated that high fructose supplementation can induce an aggressive phenotype in breast tumor cells and increase the incidence of inflammation in patients with chronic kidney disease.^22^–^24^ To fully understand the effects of dietary saccharides on cell surface glycosylation and various cellular functions, it will be helpful to elucidate the metabolic pathways of these sugars to glycoproteins.

Besides glucose, isotopically labeled monosaccharides have been used as tracers to study metabolic pathways in the cell and turnover rates of specific compounds.^25^–^30^ Earlier glycoprotein studies used radioactive isotope-labeled monosaccharides such as ^14^C-glucosamine, ^3^H-galactose and ^14^C-mannose to determine the incorporation of sugars to cell surface glycoproteins.^31^–^36^ Using these approaches, the metabolism and interconversion of common monosaccharides have been revealed, while the relative contributions of each pathway remain unknown. Moreover, the radioactive isotope-labeling methods could only provide the amount of incorporation at the total protein level. The incorporation of these monosaccharides into other groups of compounds, such as lipids, could greatly interfere with the quantitative results. More recently, labeling approaches employing stable isotope-labeled compounds and mass spectrometry detection have been developed to study the metabolism of carbohydrates and their metabolic pathways to glycoconjugates.^37^–^40^ For example, Ichikawa *et al.* studied the metabolic origins of mannose in glycoproteins by analyzing the mannose residues hydrolyzed from released *N*-glycans of 1,2-^13^C-glucose or 4-^13^C-mannose treated cells using GC-MS.^40^ Although the direct contribution of mannose to glycoprotein synthesis was estimated using this method, the incorporation of glucose and mannose into specific glycans and their parental glycoproteins were unknown. A more recent study by Xiao and Wu investigated the degradation and synthesis rates of glycoproteins using stable isotope labeling with amino acids in cell culture (SILAC) approach followed by LC-MS/MS analysis of deglycosylated peptides.^41^ This method, however, cannot be used for studying the metabolic pathways of monosaccharides and the effects of glycan composition on glycoprotein turnover.

In this study, we investigated the incorporation efficiencies and kinetics of common monosaccharides into specific glycans and glycoproteins on cell membrane by treating human intestinal and hepatic cells with ^13^C-labeled monosaccharides. Using recently developed glycomic and glycoproteomic analyses workflows,^42^ we identified the incorporation mechanisms of the different monosaccharides to specific glycans and glycoproteins using their MS and MS/MS spectra. Time-course studies using uniformly labeled ^13^C-glucose provided glycan- and protein-specific information on the turnover kinetics of cell surface glycoproteins. Incorporation levels of different saccharides in different cell lines were quantified to investigate the metabolic fates of common saccharides.

## Results and discussion

### Identification of ^13^C-Labeled glycans and glycopeptides

A workflow for the identification of ^13^C-labeled glycans and glycopeptides is shown in Fig. S1.† For each experiment, cells were cultured in either normal media with unlabeled glucose as controls or treated by media supplemented with uniformly labeled ^13^C-monosaccharides. After treatment for 3 to 96 hours with ^13^C-glucose or 72 hours with other ^13^C-labeled monosaccharides, ^13^C-isotopes were metabolically incorporated into the cell surface glycoproteins. Intact glycopeptides and released glycans from both the control and the treated samples were analyzed by LC-MS/MS as described, with the cell surface glycopeptides and glycans in the controls identified using their accurate masses and tandem MS spectra. The *N*-glycomic and glycoproteomic profiles of Caco-2 and M213 cells presented in this work have also been obtained previously.^42^ The retention times and monoisotopic peaks in both control and labeled compounds were used to identify the labeled species. By comparing the isotope distributions of each compound from the control and the treated samples, ^13^C-labeled glycans and glycopeptides were identified and further confirmed by their respective tandem MS spectra.

### Elucidation of isotopic incorporation

**Fig. 1:**
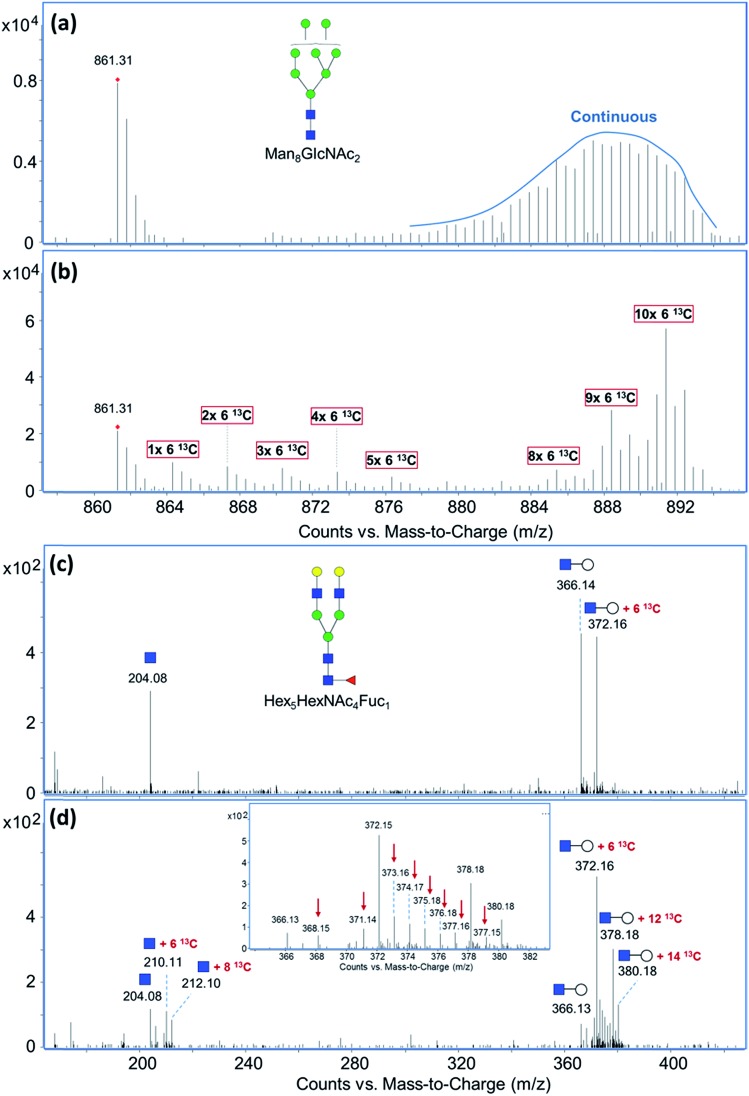
MS and MS/MS spectra for the elucidation of incorporation mechanisms. (a) MS spectrum of *N*-glycan Man_8_GlcNAc_2_ from ^13^C-fructose-treated Caco-2 cell. (b) MS spectrum of *N*-glycan Man_8_GlcNAc_2_ from ^13^C-fructose-treated M213 cell. (c) MS/MS spectrum of *N*-glycan Hex_5_HexNAc_4_Fuc_1_ (precursor *m*/*z* 897.35) from ^13^C-galactose-treated M213 cell. (d) MS/MS spectrum of *N*-glycan Hex_5_HexNAc_4_Fuc_1_ (precursor *m*/*z* 919.41) from ^13^C-mannose-treated M213 cell.

Exogenous dietary monosaccharides such as glucose, galactose, mannose, and fructose can be transported across the plasma membrane into the cell and converted to their phosphorylated forms (Fig. S2†) before they participate in glycoprotein synthesis *via* three different pathways: glycolysis, salvage or direct activation to the same sugar donors, and conversion to other activated sugar donors. The first pathway (glycolysis) will result in an increase in the molecular weights of glycans and glycopeptides by different mass units (*M* + *x*) while the latter two pathways will incorporate intact monosaccharides resulting in a mass increase in multiples of six (*M* + *n* × 6). The three incorporation mechanisms can be elucidated by the MS and MS/MS spectra of glycans and glycopeptides with ^13^C incorporation. Fig. 1a and b shows an example of the differential incorporation mechanisms revealed by the mass spectra of a high mannose type *N*-glycan, Man_8_GlcNAc_2_, detected in ^13^C-fructose-treated Caco-2, an epithelial colorectal adenocarcinoma cell line, and M213, an intrahepatic cholangiocarcinoma cell line, respectively. In the intestinal epithelial cell, the ^13^C-incorporated Man_8_GlcNAc_2_ had a continuous isotope distribution ranging from *m*/*z* 874 to *m*/*z* 893, corresponding to the incorporation of ^13^C into approximately 26 to 64 of carbons in this glycan. The continuous and uniform distribution demonstrated incorporation through glycolysis. In the hepatic M213 cell, however, a combination of continuous and discrete isotope distribution ranging from *m*/*z* 864 to *m*/*z* 891, with mass increments of six (*M* + *n* × 6) was observed for this *N*-glycan indicating the intact conversion of fructose into mannose and GlcNAc residues. These results demonstrated the differential utility of fructose in cells from different tissues.

More detailed information regarding the incorporation pathways were obtained from MS/MS spectra of the specific glycans labeled with ^13^C. Fig. 1c shows the MS/MS spectrum of a fucosylated complex-type *N*-glycan, Hex_5_HexNAc_4_Fuc_1_, from ^13^C-galactose-treated M213 cell with a precursor *m*/*z* of 897.35. Compared to its monoisotopic molecular ion with a *m*/*z* of 894.34 (*z* = 2), six carbons were ^13^C-labeled. The MS/MS spectrum containing fragment ions with *m*/*z* 204.08 (HexNAc), 366.14 (HexNAc + Hex), and 372.16 (HexNAc + Hex + 6 ^13^C) revealed that all of the labeled carbons were on a hexose residue. Because ^13^C-galactose was only slightly incorporated into the high mannose glycan from the same M213 cell sample, we concluded that ^13^C-galactose was mainly utilized through direct incorporation as an intact galactose residue in hepatic cell. In contrast, when M213 cells were treated with ^13^C-mannose, fragment ions with both increments of six (*m*/*z* 210.11, HexNAc + 6 ^13^C; *m*/*z* 372.16, HexNAc + Hex + 6 ^13^C; *m*/*z* 378.18, HexNAc + Hex + 12 ^13^C) corresponding to intact incorporation and increments of one (indicated by red arrows in Fig. 1d) corresponding to incorporation by glycolysis were observed for glycan composition Hex_5_HexNAc_4_Fuc_1_ with *m*/*z* 919.41 (50 carbons incorporated with ^13^C) (Fig. 1d). The comparison and combination of information from MS and MS/MS spectra provided extensive elucidation of incorporation pathways for saccharides from different sources and cell lines of different tissues.

### Quantitation of incorporation efficiency

**Fig. 2:**
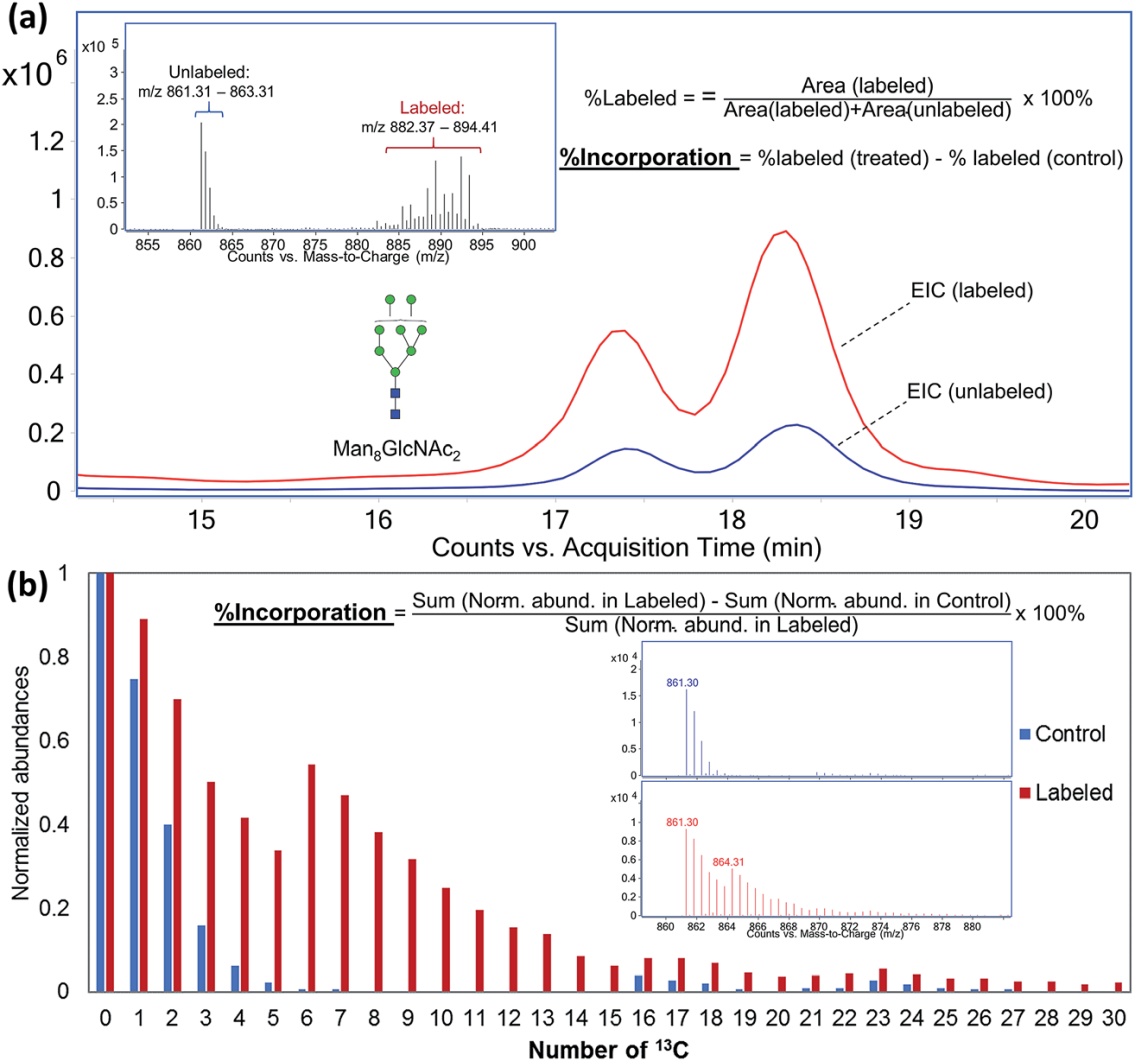
Two approaches for the quantitation of incorporation efficiency. (a) Quantitation using the peak areas obtain from the EICs of the same compound from labeled and unlabeled samples. (b) Quantitation using the summed normalized abundances of the same compound from labeled and unlabeled samples.

To compare the incorporation behavior and metabolic pathways associated with different ^13^C-labeled saccharides in different cell lines, a method for quantitative measurement of incorporation efficiency is necessary. As illustrated in Fig. 2, two quantitation approaches were used in this study depending on the extent of ^13^C-incorporation. In the first method where most of the carbons in the glycan or glycopeptide were labeled by ^13^C and the isotope distribution of the labeled compound had no overlap with its original isotopic peaks (Fig. 2a), chromatograms were extracted for the *m*/*z* ranges with and without labeling. Peak areas obtained by manual integration of the EICs were used to calculate the percentage of ^13^C-labeled compounds. Fractional incorporation was then obtained by subtracting the (%)-labeled value of the control sample from that of the treated sample to correct the error caused by possible noise peaks. This approach, however, is less accurate when the isotopic distributions of the labeled and unlabeled compounds overlap. Consequently, the averaged isotope distributions obtained from MS spectra of the same compound were compared between the control and the treated samples after all the isotopes were normalized to their respective monoisotopic peaks (Fig. 2b). By comparing the summed normalized abundances of all isotopes from the two samples, the percent incorporation is determined more accurately.

### Time-course study to obtain incorporation kinetics

**Fig. 3:**
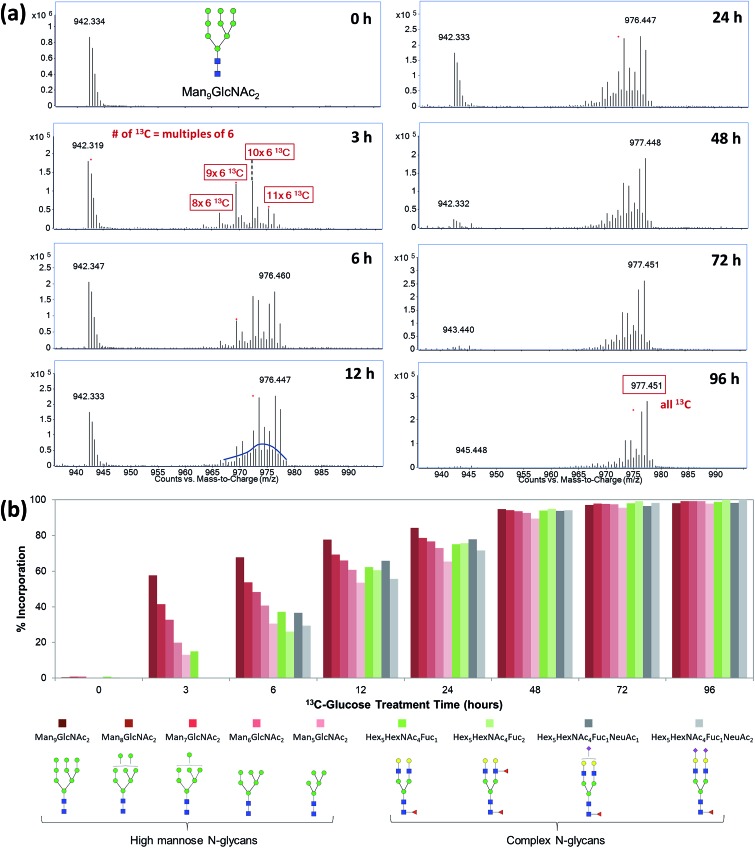
Time-course study of incorporation kinetics of cell surface glycans. (a) MS spectra of *N*-glycan Man_9_GlcNAc_2_ from M213 cell after 3 to 96 hours of treatment with ^13^C-glucose. (b) Rates of ^13^C-incorporation into different types of *N*-glycans.

Although it was shown that each cell line has a unique and essentially constant glycosylation profile until they were subcultured at higher passages,^16^,^43^ the cell surface glycoproteins are known to be constantly renewing in living cells. Factors such as the structures of attached glycoforms and the interactions between glycoprotein and ligands can greatly affect the stability and turnover rate of a glycoprotein.^44^ By profiling the incorporation kinetics of uniformly labeled ^13^C-glucose to cell surface glycans and glycoproteins through a time-course study, we can elucidate the turnover rates of different glycoproteins and glycan structures. Fig. 3a shows the incorporation of ^13^C-glucose into a high mannose type *N*-glycan, Man_9_GlcNAc_2_, from hepatic M213 cell, after 3 to 96 hours of treatment. The incorporation level of this glycan was found to be approximately 60% after the cell was cultured in ^13^C-glucose-containing medium for only three hours, indicating the rapid turnover rates of membrane glycoproteins with this glycoform. MS spectra from different time points revealed that the dominant incorporation mechanism of ^13^C-glucose during the first few hours was intact conversion because the isotope distribution showed distinct mass increments of six. Starting from approximately 12 hours, however, glycolysis becomes the major incorporation pathway. After 72 to 96 hours of treatment, the ^13^C-incorporation efficiency reached 100%, and the ion with *m*/*z* 977.45, corresponding to Man_9_GlcNAc_2_ with all 70 carbons isotopically labeled was the highest peak.

To evaluate and compare the turnover rates of different glycan types, we calculated the incorporation rates of five high-mannose-type *N*-glycans containing two GlcNAc residues and five to nine mannose residues, two fucosylated-only complex type *N*-glycans, Hex_5_HexNAc_4_Fuc_1_ and Hex_5_HexNAc_4_Fuc_2_, and two fucosylated and sialylated complex type *N*-glycans, Hex_5_HexNAc_4_Fuc_1_NeuAc_1_ and Hex_5_HexNAc_4_Fuc_1_NeuAc_2_, from M213 cell. The incorporation rates of ^13^C-glucose into various glycans were graphed against treatment time for the cell (Fig. 3b). In general, the turnover rates of different types of *N*-glycans were consistent with their synthesis and maturation pathways.^45^–^47^ The fastest rate of incorporation within this group was for Man_9_GlcNAc_2_ which is the glycan synthesized in the earliest part of the glycosylation process, while still in the endoplasmic reticulum (ER). The fast rate of incorporation means high turnover rate due to both the high synthesis rate of the glycan and perhaps the rapid degradation rate of the attached proteins. While another high mannose *N*-glycan, Man_8_GlcNAc_2_, is also produced in the ER, but it had a slightly lower turnover rate due potentially to the extra enzymatic processing step by α-mannosidase I. Similarly, the other high mannose glycans, Man_7_GlcNAc_2_, Man_6_GlcNAc_2_, and Man_5_GlcNAc_2_ had decreasing turnover rates. In general, the high mannose type glycans turned over within the first three hours, significantly faster than the fucosylated and sialylated complex type glycans. The latter groups are modified in the Golgi and therefore require more enzymatic processing steps. The lower turnover rates of sialylated glycans were also consistent with previous findings that terminally charged glycans can extend the circulatory half-life of glycoproteins.^48^ Nonetheless, the incorporation levels of all the glycans reached >90% after 48 hours and approximately 100% after 72 hours, indicating all the cell surface glycoproteins were turned over.

**Fig. 4:**
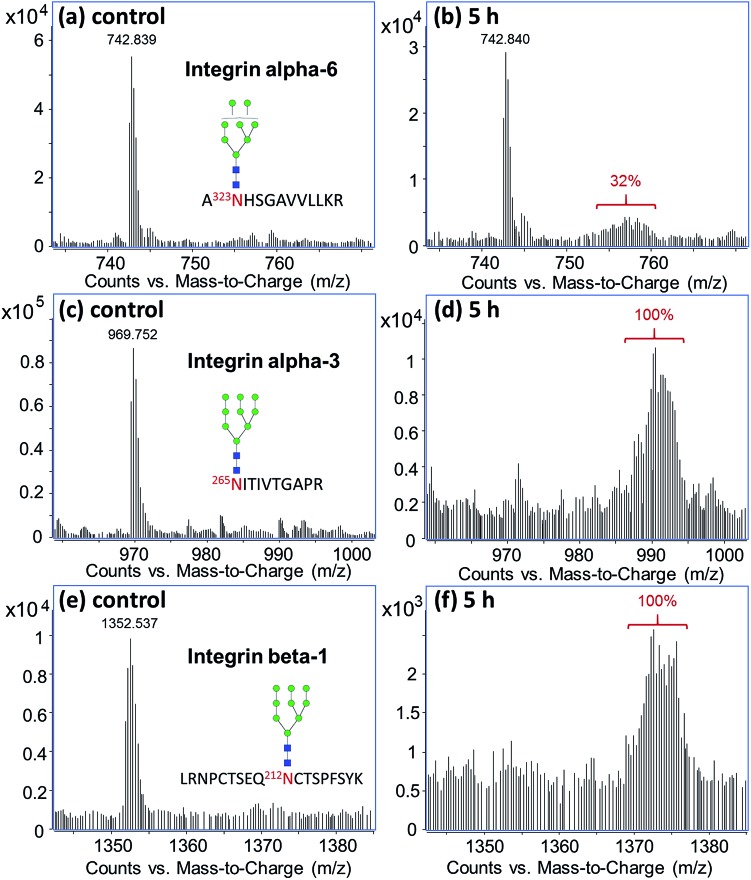
^13^C-incorporation into integrin glycopeptides from M213 cell treated with ^13^C-glucose for five hours.

Glycoprotein turnover rates are closely related to glycan types but can vary more with protein structures, functions, and localization. With comprehensive glycoproteomic analysis of the untreated cells, we can profile the turnover rates of specific glycoproteins. For example, integrins are a group of cell surface glycoproteins that play important parts in cell–cell adhesion and intracellular signaling.^11^,^49^ Each integrin consists of an alpha and a beta subunit that are both highly glycosylated.^50^,^51^ Fig. 4 shows the differential turnover rates of three glycopeptides from different integrin subunits, α6, α3, and β1, found on M213 cell. After five hours of treatment with ^13^C-glucose, both the α3 and β1 subunits were 100% exchanged, while subunit α6 was only 32% labeled although all three glycopeptides carried a high mannose type *N*-glycan. These results showed that the α3 and β1 subunits in M213 cell have similar metabolic behavior, consistent with the two subunits being in the same integrin protein complex. The isotopic distribution for the α3 and β1 subunits spanned up to around *m*/*z* 994 and 1377, respectively, corresponding to a total of ∼75 labeled carbons in the glycopeptides. Compared to the incorporation profile of the glycan alone (Fig. 3a) where the majority of the 70 carbons from Man_9_GlcNAc_2_ were labeled after six hours, the glycopeptide profiles showed that the ^13^C-incorporation occurred mainly on the glycan side accompanied with minor incorporation on the polypeptide.

### Differential metabolic fates of monosaccharides in various cell lines

**Fig. 5:**
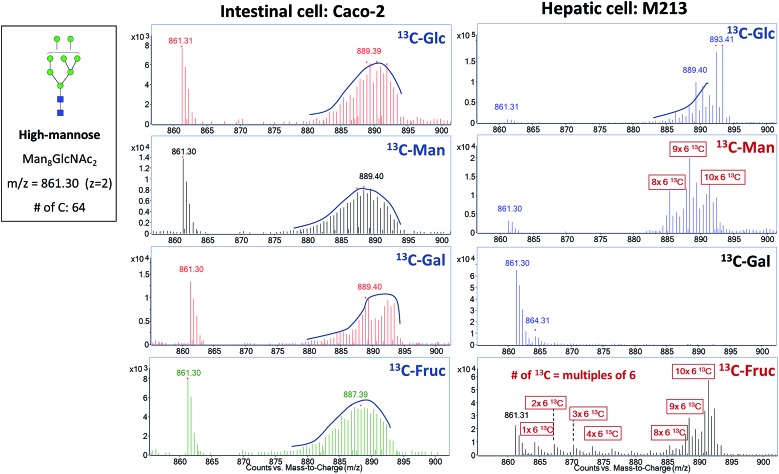
Incorporation of ^13^C-labeled glucose, mannose, galactose, and fructose into a high mannose type *N*-glycan from Caco-2 and M213 cells.

With the qualitative and quantitative methods described above to characterize the incorporation mechanisms and efficiencies, we studied the incorporation behavior of four common dietary monosaccharides, glucose, galactose, fructose, and mannose, in different cell lines. MS spectra of two *N*-glycans, Man_8_GlcNAc_2_ and Hex_5_HexNAc_4_Fuc_1_NeuAc_1_, from intestinal Caco-2 cells and hepatic M213 cells after treatment with these four ^13^C-labeled monosaccharides are shown in Fig. 5 and S3.† Using the MS spectra and EICs of labeled and unlabeled compounds, the incorporation levels as well as the averaged percentage of ^13^C-replaced carbons were calculated as shown in Table 1. As the major energy and carbon source for most organisms, glucose was efficiently incorporated into both glycan types from both cell lines through the glycolysis pathway. MS/MS spectrum of a fucosylated and sialylated *N*-glycan, Hex_5_HexNAc_5_Fuc_1_NeuAc_2_, demonstrated that glucose could be efficiently converted to any type of monosaccharides including mannose, GlcNAc, galactose, fucose, and sialic acid (Fig. S4a†). Exogenous fructose and mannose, after conversion to fructose-6-P and mannose-6-P, respectively, can also be converted to all other sugars donors in the cell.^34^,^52^ Their incorporation mechanisms, however, were found to be different between intestinal Caco-2 and hepatic M213 cells. In intestinal cells, the glycolysis pathway was dominant for both monosaccharides. While in hepatic cells, direct incorporation of mannose into high mannose *N*-glycans and the intact conversion of fructose to mannose became more prominent. (Fig. S4b and S5†) The intact conversion of mannose to galactose, GlcNAc, and fucose was also found to be more efficient than its conversion to sialic acid. Despite the differential metabolic pathways, the incorporation levels of fructose and mannose remained high in both cell lines when no glucose was provided. However, when the cells were supplemented with both ^13^C-fructose and unlabeled glucose, the incorporation of fructose dramatically decreased for both cells even though the concentration of fructose was much higher than glucose (25 mM *vs.* 10 mM). (Fig. S6†) Similar results were observed for ^13^C-mannose, demonstrating the preferential utilization of glucose by the cell as a carbon source.

**Table 1:** Comparison of incorporation mechanisms and efficiencies of uniformly labeled (UL) ^13^C-monosaccharides in intestinal Caco-2 and hepatic KKU-M213 cell lines

^13^C-Saccharide	Incorporation mechanism		% Incorporation				% (Number of ^13^C/total number of C)			
			Man_8_GlcNAc_2_		Hex_5_HexNAc_4_Fuc_1_NeuAc_1_		Man_8_GlcNAc_2_		Hex_5_HexNAc_4_Fuc_1_NeuAc_1_	
	Caco-2	M213	Caco-2	M213	Caco-2	M213	Caco-2	M213	Caco-2	M213
UL-^13^C-Glc	Glycolysis	Glycolysis	86%	98%	86%	97%	91%	97%	86%	95%
UL-^13^C-Man	Glycolysis	Glycolysis & intact	82%	95%	78%	97%	88%	84%	72%	68%
UL-^13^C-Gal	Glycolysis	Intact	80%	37%	75%	82%	88%	9%	80%	15%
UL-^13^C-Fruc	Glycolysis	Glycolysis & intact	84%	91%	76%	92%	86%	86%	72%	83%

More distinct features of incorporation were observed for ^13^C-galactose-treated cells. While in Caco-2 cell it was incorporated in the same way as the other three monosaccharides through the glycolysis pathway, it could only be utilized as an intact residue in M213 cells. For the high mannose type *N*-glycan where there is no galactose residue, the incorporation level was only 37% with six carbons replaced by ^13^C (Table 1). For the complex type *N*-glycan with two galactose residues, however, incorporation of six or twelve ^13^C was observed with a total incorporation level of 82%, demonstrating the direct incorporation of galactose in the hepatic cell.

## Discussion

In this study, we present a stable isotope-labeling method to elucidate the incorporation of exogenous monosaccharides to cell membrane glycoproteins and evaluate the utilization efficiencies of different saccharides thereby yielding their relative contributions in different metabolic pathways. These glycomic and glycoproteomic approaches are providing previously unobtainable information regarding monosaccharide utilization in the formation of glycoproteins. By combining metabolic isotope-labeling of glycoproteins with glycomic and glycoproteomic analyses by LC-MS/MS, we were able to unveil the metabolic origins and turnover kinetics of specific glycans and glycoproteins on the cell membrane. The results showed glycan- and protein-specific turnover rates of membrane glycoproteins and unique ^13^C-incorporation patterns of different saccharides in intestinal and hepatic cells. This stable isotope-labeling method is readily applicable to *in vivo* studies to investigate tissue-specific utilization of dietary saccharides. In combination with studies on the functions of different glycosylation patterns, this method will provide valuable guidance for dietary manipulation of glycosylation and fate mapping of exogenous supplements.

Dietary saccharides are not only an essential energy source but also the major carbon source of the cell. Small differences in the structure of exogenous monosaccharides can have a significant impact on cell glycosylation and potentially function. Understanding the metabolic pathways of dietary saccharides for incorporating into membrane glycoproteins may be necessary for controlling glycosylation and avoiding possible disorders caused by diet. Earlier advancements in glycobiology have revealed the metabolic pathways of common monosaccharides for the synthesis of nucleotide sugar donors, the critical precursors for glycosylation.^12^ However, less is known regarding the relative contributions of the pathways in different organisms. For example, glucose and mannose are both used in a multitude of glycan structures. Their phosphate forms, Glc-6-phosphate and Man-6-phosphate are the central molecules in the monosaccharide biosynthesis pathways and can be converted to all other sugars. The results presented here indicate that fucosylated and sialylated *N*-glycans employ glucose and mannose through the glycolysis pathways at least in the cell lines studied here. However, with *N*-glycans that contain multiple mannose residues as in high mannose glycans, intact incorporation of exogenous mannose was significant in M213 cells. The cell-dependent incorporation of monosaccharides was also demonstrated for galactose and fructose, where in the intestinal epithelial cell glycolysis is always the dominant pathway while in the hepatic cell more intact incorporation was observed.

The monosaccharides included in these studies can represent different components of diet. Glucose is found primarily as starch, while fructose is found in fruits and vegetables. Mannose is found in all types of glycoprotein-containing food products and galactose is a monosaccharide component of lactose from dairy products.^53^ While this notion is simplistic, it does point to variations in utilization of various monosaccharides from diet that is both glycoconjugate- and tissue-specific. Furthermore, we have previously shown that feeding cells with different monosaccharides can alter the membrane glycosylation.^16^ The implications are that diet can alter the membrane glycosylation and therefore the cellular function. Indeed, cell surface glycosylation can alter the migratory properties of cells, its ability to protect itself from infection, and its ability to migrate.^16^,^54^,^55^ We can therefore infer that diet can affect the fundamental properties of cell.

## Experimental section

### Cell culture

Human colorectal adenocarcinoma Caco-2 cells were obtained from American Type Culture Collection (ATCC, VA) and grown in Eagle's Minimum Essential Medium (EMEM) supplemented with non-essential amino acids and 2 mM l-glutamine. Human cholangiocarcinoma KKU-M213 cells were obtained from the Japanese Collection of Research Bioresources Cell Bank (JCRB, Osaka, Japan) and grown in Ham's F12 medium. All the media were supplemented with 10% (v/v) fetal bovine serum and 100 U mL^–1^ penicillin and streptomycin. Cells were subcultured at 80% confluency and maintained at 37 °C in a humidified incubator with 5% CO_2_.

### Isotope labeling of cell surface glycoproteins

For isotope labeling experiments, all the cells were cultured in Dulbecco's Modified Eagle's Medium (DMEM) containing 25 mM glucose for 24 h before treated with glucose-free DMEM supplemented with specific ^13^C-labeled monosaccharides (Omicron Biochemicals, IN) at concentration of 25 mM for 72 h unless specified otherwise. Controls were cultured in DMEM containing 25 mM glucose for the same amount of time before both the control and treated cells were harvested by scraping. For the time-course study using 25 mM of uniformly labeled glucose (^13^C_6_-glucose), cells were washed three times with PBS and harvested by scraping after 3, 6, 12, 24, 48, 72, and 96 h of treatment.

### Cell membrane extraction

Extraction of the cell membrane fraction was performed as described previously with modified procedures.^43^,^56^ In brief, harvested cells were resuspended in homogenization buffer containing 0.25 M sucrose, 20 mM HEPES-KOH (pH 7.4), and 1 : 100 protease inhibitor (EMD Millipore, CA). Cell lysis was performed on ice using a probe sonicator (Qsonica, CT) with five alternating on and off pulses in 5 and 10 s intervals, respectively. Whole cell lysates were centrifuged at 2000×*g* for 10 min to remove the nuclear fractions and cellular debris. The supernatant was collected for ultracentrifugation at 200 000×*g* for 45 min at 4 °C. The pellet was resuspended and repelleted by ultracentrifugation using the same conditions in 0.2 M Na_2_CO_3_ (pH 11) followed by water to fragment the endoplasmic reticulum and remove the cytoplasmic fraction, respectively. The resulting membrane fraction was isolated and stored at –20 °C until further processing.

### Enzymatic release and purification of *N*-Glycans

Membrane pellets were resuspended in 100 μL of 100 mM ammonium bicarbonate with 5 mM dithiothreitol and heated for 10 s at 100 °C to thermally denature the proteins. To release the *N*-glycans from membrane proteins, 2 μL of peptide *N*-glycosidase F (New England Biolabs, MA) were added to the samples and incubated at 37 °C in a microwave reactor (CEM Corporation, NC) for 10 min at 20 watts. After addition of 350 μL of nanopore water, samples were ultracentrifuged at 200 000×*g* for 45 min at 4 °C to precipitate the membrane fractions with lipids and residual deglycosylated proteins. The supernatant containing the released *N*-glycans was collected. *N*-Glycans were purified by solid phase extraction containing a porous graphitized carbon (PGC) matrix (Grace, IL). Eluted fractions were dried *in vacuo* before they were reconstituted in 30 μL of nanopore water and analyzed by LC-MS/MS.

### Glycomic analysis by LC-MS/MS

For *N*-glycan analysis, 5 μL of each reconstituted sample was injected to an Agilent nanoLC/ESI-QTOF-MS system (Agilent Technologies, CA) equipped with a microfluidic chip, which consists of an enrichment and an analytical column both packed with porous graphitized carbon. A binary gradient was applied to separate and elute glycans at a flow rate of 0.3 μL min^–1^ using solvents (A) 3% (v/v) acetonitrile and 0.1% (v/v) formic acid in water and (B) 90% (v/v) acetonitrile in 1% (v/v) formic acid in water. MS spectra were acquired at 1.5 s per spectrum over a mass range of *m*/*z* 600–2000 in positive ionization mode. Collision-induced dissociation (CID) was performed with nitrogen gas using a series of collision energies (*V*_collision_) dependent on the *m*/*z* values of the *N*-glycans, based on the equation: *V*_collision_ = *m*/*z* (1.8/100 Da) V – 2.4 V.

### Glycomic data analysis

*N*-Glycans in the control samples were identified with an in-house library using MassHunter Qualitative Analysis B.06.01 (Agilent Technologies, CA). Deconvoluted masses were compared to the theoretical masses using a mass tolerance of 10 ppm. Area under the peak was used to represent the abundance of each glycan. Tandem MS spectra were used to confirm the compositions and putative structures of the glycans.

### Preparation of glycopeptides

Portions of the extracted plasma membrane fractions were suspended in 60 μL of 8 M urea, reduced with 18 mM dithiothreitol at 55 °C for 50 min, alkylated with 27 mM iodoacetamide at room temperature for 30 min, diluted to 1 M urea with 50 mM ammonium bicarbonate, and incubated with 2 μg trypsin at 37 °C overnight. The resulting glycopeptides were enriched by solid-phased extraction using iSPE-HILIC cartridges and dried *in vacuo*.

### Glycoproteomic analysis by LC-MS/MS

Glycopeptides were dissolved in 2% (v/v) acetonitrile and 0.1% (v/v) trifluoroacetic acid in water and separated using a reverse-phase Michrom Magic C18 AQ column (200 μm, 150 mm) coupled with a Q Exactive Plus mass spectrometer through a Proxeon nano-spray source (Thermo Scientific, CA). A binary gradient was applied at a constant flow rate of 300 nl min^–1^ using 0.1% (v/v) formic acid in (A) water and (B) 100% acetonitrile. MS spectra were acquired over a mass range of *m*/*z* 350–1600 in positive ionization mode. Higher-energy collisional dissociation (HCD) with stepped collision energies (17, 27, 37) was performed in data-dependent acquisition mode.

### Glycoproteomic data analysis

Glycoproteins and glycopeptides were identified from the tandem mass spectra using Byonic software (Protein Metrics, CA) against the reviewed Swiss-Prot human protein database (20 207 entries) with sample-specific parameters as determined from Byonic Preview (Protein Metrics, CA): mass tolerances of 5–10 ppm for the precursor and 10–20 ppm for fragment ions; peptide probability >0.95; carbamidomethylation of cysteine as a fixed modification; oxidation of methionine and tryptophan, deamidation of asparagine and glutamine, acetylation of the protein *N*-terminus, and ammonia loss of cysteine as variable modifications; *N*-glycosylation of asparagine (in-house human database of 369 entries); two missed cleavage sites. Identifications were filtered with a 1% false discovery rate and were accepted if their Log[Prob] values were greater than 2.

## Author contributions

Conceptualization, G. X., D. P. and C. B. L.; methodology, G. X. and D. P.; investigation, G. X., M. Y. W., Q. L. and Z. C.; writing – original draft, G. X. and C. B. L.; writing – review & editing, G. X., M. Y. W., Q. L., D. P. and C. B. L.; funding acquisition, C. B. L.; supervision, C. B. L. All authors proofread and commented on the manuscript.

## Conflicts of interest

The authors declare no competing interests.

## Supplementary Material

Supplementary information
