# Supplementary material for: Unveiling the metabolic fate of monosaccharides in cell membranes with glycomic and glycoproteomic analyses
Source: Chem Sci. 2019 Jun 11;10(29):6992–7002. doi: 10.1039/c9sc01653h (PMC6676465; doi:10.1039/c9sc01653h)

Electronic Supplementary Material (ESI) for Chemical Science.
This journal is © The Royal Society of Chemistry 2019

Supplementary Data

Figure S1. Incorporation of ^{13}C-saccharides to cell membrane glycoproteins and identification of ^{13}C-labeled glycans and glycopeptides.

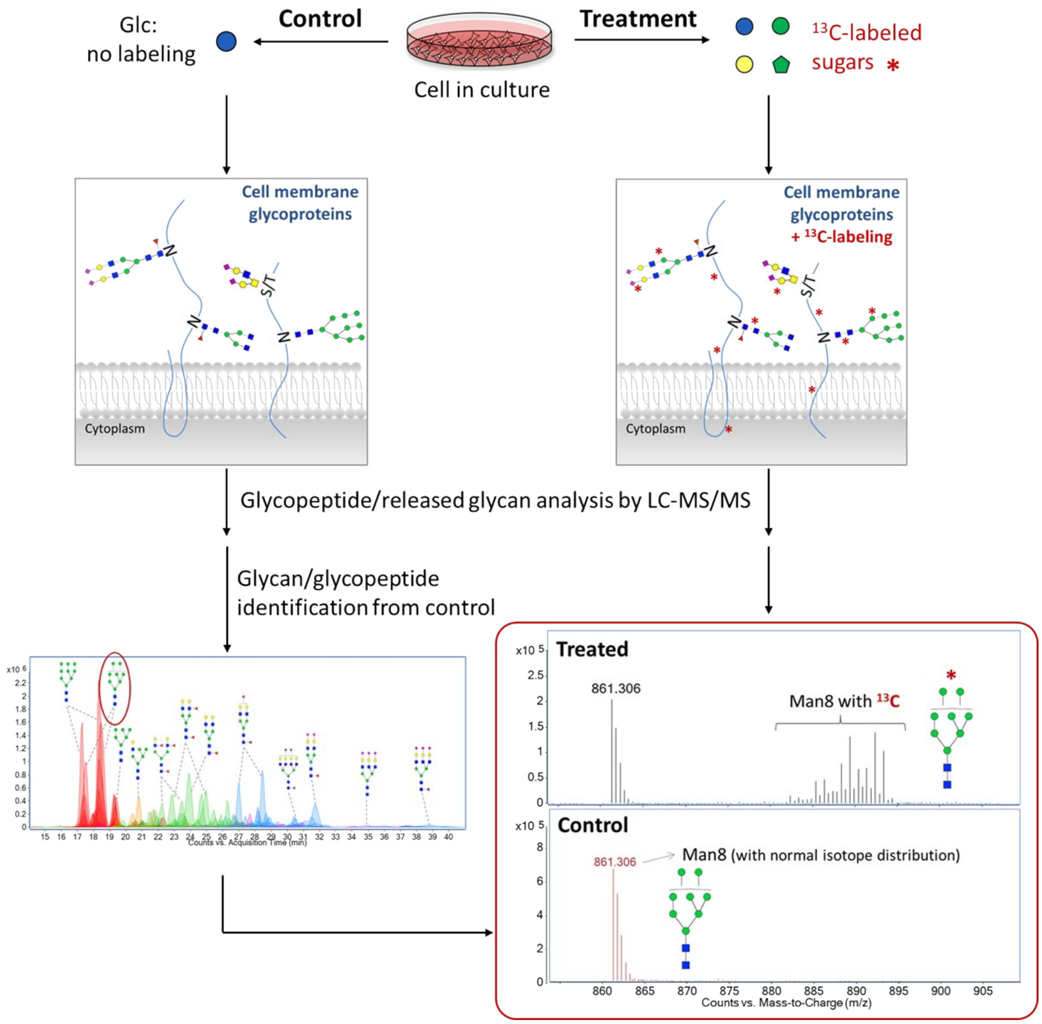

Figure S2. Different mechanisms for the incorporation of monosaccharides to glycoproteins.

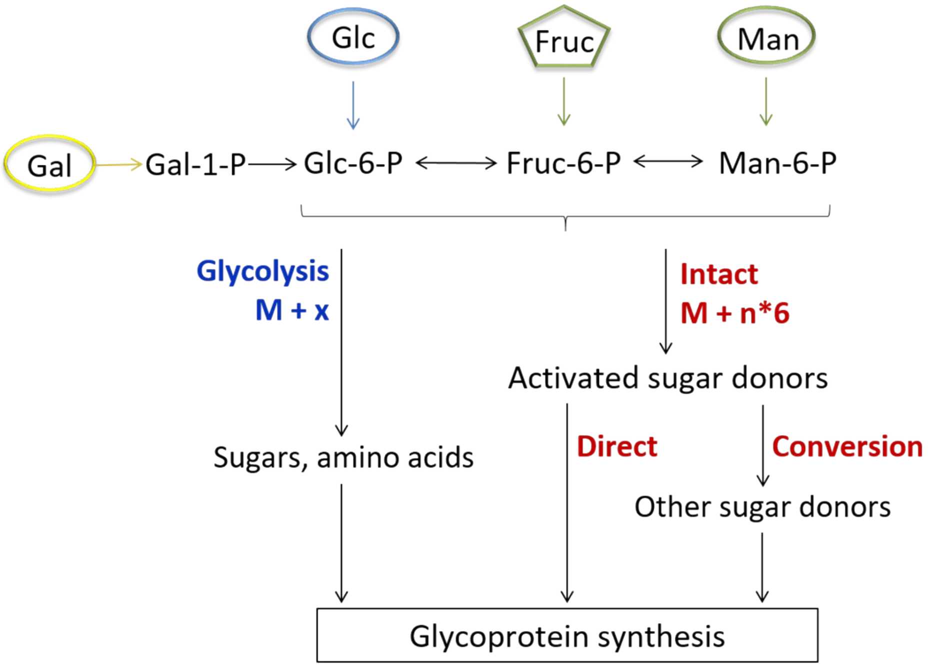

Figure S3. Incorporation of ^{13}C-labeled glucose, mannose, galactose, and fructose into a fucosylated and sialylated complex-type N-glycan from Caco-2 and M213 cells.

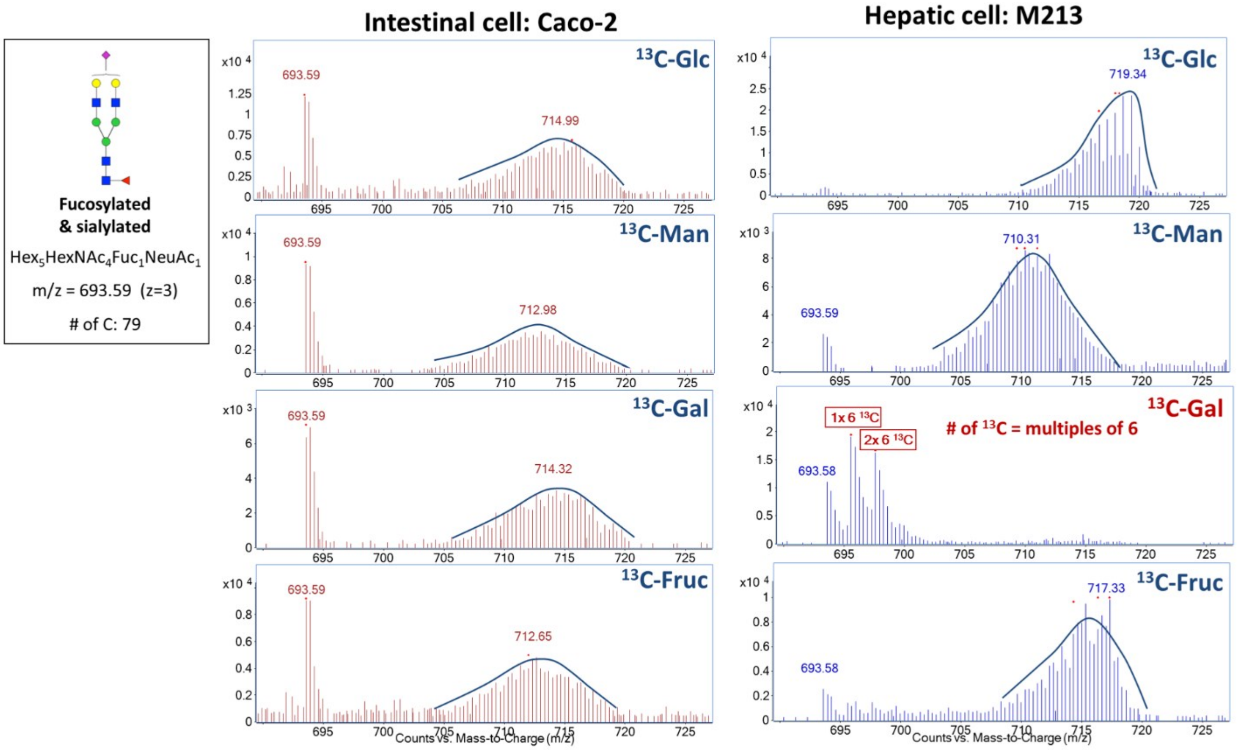

Figure S4. MS/MS spectra illustrating the incorporation and interconversion pathways of (a) ^{13}C-labeled glucose in Caco-2 cells; (b) ^{13}C-labeled mannose in M213 cells.

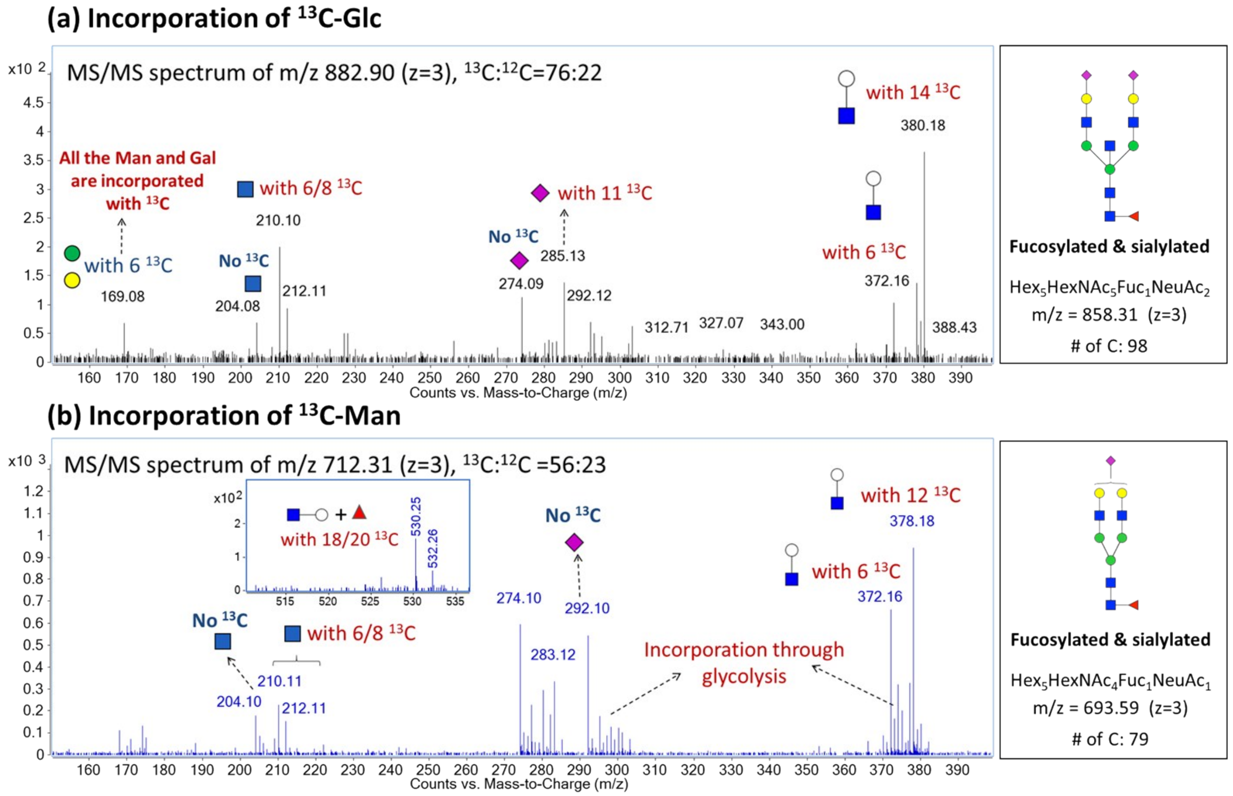

Figure S5. MS/MS spectra illustrating the differential incorporation pathways of ^{13}C-labeled fructose in intestinal Caco-2 cell and hepatic M213 cell.

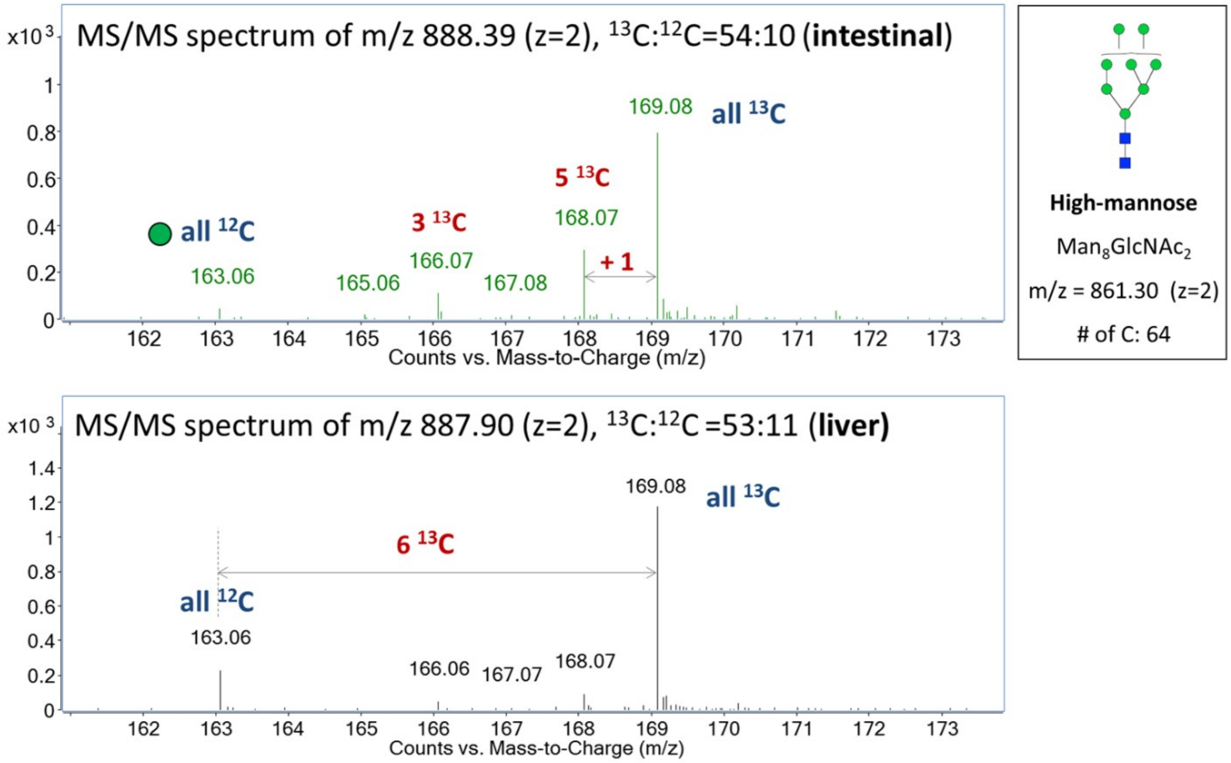

Figure S6. Decreased incorporation levels of ^{13}C-fructose when glucose is present.

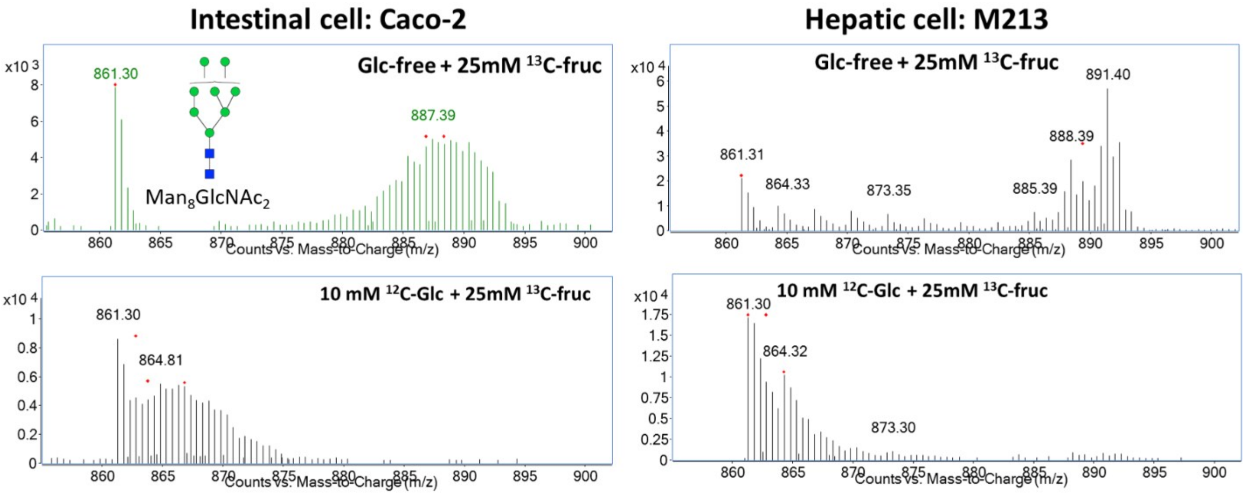

Supplement: Supplementary file 1 [file SC-010-C9SC01653H-s001.pdf]
